# Optimization of the Extraction of Proanthocyanidins from Grape Seeds Using Ultrasonication-Assisted Aqueous Ethanol and Evaluation of Anti-Steatosis Activity In Vitro

**DOI:** 10.3390/molecules27041363

**Published:** 2022-02-17

**Authors:** Wasitha P. D. W. Thilakarathna, H. P. Vasantha Rupasinghe

**Affiliations:** Department of Plant, Food, and Environmental Sciences, Faculty of Agriculture, Dalhousie University, Truro, NS B2N 5E3, Canada; wasitha@dal.ca

**Keywords:** steatosis, flavonoids, grapes, by-products, green extraction, condensed tannins, ultrasonication

## Abstract

Conventional extraction methods of proanthocyanidins (PAC) are based on toxic organic solvents, which can raise concerns about the use of extracts in supplemented food and nutraceuticals. Thus, a PAC extraction method was developed for grape seeds (GS) and grape seed powder using food-grade ethanol by optimizing the extraction conditions to generate the maximum yield of PAC. Extraction parameters, % ethanol, solvent: solid (s:s) ratio, sonication time, and temperature were optimized by the central composite design of the response surface method. The yields of PAC under different extraction conditions were quantified by the methylcellulose precipitable tannin assay. The final optimum conditions were 47% ethanol, 10:1 s:s ratio (*v:w*), 53 min sonication time, and 60 °C extraction temperature. High-performance liquid chromatography analysis revealed the presence of catechin, procyanidin B2, oligomeric and polymeric PAC in the grape seed-proanthocyanidin extracts (GS-PAC). GS-PAC significantly reduced reactive oxygen species and lipid accumulation in the palmitic-acid-induced mouse hepatocytes (AML12) model of steatosis. About 50% of the PAC of the GS was found to be retained in the by-product of wine fermentation. Therefore, the developed ethanol-based extraction method is suitable to produce PAC-rich functional ingredients from grape by-products to be used in supplemented food and nutraceuticals.

## 1. Introduction

Proanthocyanidins (PAC), also known as condensed tannins, are the oligomers or polymers of flavan-3-ol molecules linked together through interflavan linkages. Oligomeric and polymeric PAC predominantly consist of catechin and epicatechin monomers [1]. Monomers of gallocatechin, epigallocatechin, afzelechin, and epiafzelechin are also present in the molecular structure of some PAC [2]. PAC are ubiquitously found in plant-based food, including berries, nuts, cereals, beans, and their products [3]. PAC can be used as a food additive to impart astringency, microbial stability, foamability, oxidative stability, and heat stability to food products [4].

The interest in PAC has considerably increased over time with the discovery of the potential beneficial physiological function of PAC [5,6]. PAC are excellent antioxidant and anti-inflammatory molecules [7] capable of influencing cell-signaling pathways [8]. The potential of PAC to alleviate chronic metabolic diseases has been shown by a number of studies. Supplementation of PAC has reduced obesity and obesity-associated complications by improving the blood lipid profile [9], intestinal microflora [10], and interfering adipogenesis [11]. However, the potential of PAC ingestion to reduce the body weight is still controversial [12]. The ability of PAC to improve vascular endothelial function and improve blood lipid profile by reducing triglyceride and low-density lipoprotein cholesterol levels is beneficial in reducing the risk of hypertension and cardiovascular diseases [13]. PAC also depict anticancer properties, especially in colorectal cancers [14]. PAC can promote cancer cell death [15] and inhibit angiogenesis and cancer metastasis [16,17]. However, the bioavailability of oligomeric and polymeric PAC depends on their bioconversion by the colonic microbiota. The microbial metabolites of PAC are proven to possess anticancer properties [18]. The interdependency of PAC and bioconverting microbes can open a new field of study to develop PAC-based synbiotics with health benefits [19].

Grape seeds (GS) are an excellent source of PAC (around 35 mg/g dry seeds [20]) and a widely available, underutilized by-product of grape juice and wine processing [3]. Grape seed-proanthocyanidin (GS-PAC) is a heterogeneous mixture of 5–30% monomers, 17–63% oligomers, and 11–39% polymers [21]. A number of PAC isolation methods of GS, such as single and multiple solvent extractions aided by ultrasound, microwave, enzymatic, and mechanical treatment as well as liquid/liquid phase separation, have been reported [22,23,24,25]. Ultrasonication can be coupled with many conventional PAC extraction methods to enhance extraction efficiency [26]. When plant materials are used as the source of bioactive, ultrasonication can be applied to destroy the cell walls to enable better interaction of extraction solvents with phytochemicals. Moreover, application of ultrasonication has been proven to increase extraction efficiency at low temperatures [27], which is significantly important to prevent the thermal degradation of phytochemicals [28]. Many of the recently reported liquid/liquid-based proanthocyanidin extraction methods use aqueous organic solutions (e.g., acetone, methanol, and ethanol) as the extraction solvent [29,30,31]. These aqueous two-phase extraction (ATPE) methods can use ionic liquids (salt solutions) to increase the efficiency of proanthocyanidin extraction [31]. Despite the high extraction efficiencies and low impact on environment, scalability of ATPE methods to a commercial level remains controversial due to the complexity of phase separation procedures, solvent recovery, and operational costs [32]. Subcritical water [33] and supercritical CO_2_ extraction [34] methods are also among the tested PAC extraction methods. Ethyl acetate, acetone, hexane, methanol, ethanol, and water are the most commonly used solvents to extract PAC [35,36]. However, ethanol is preferred over other solvents for the extraction of plant bioactives due to its lesser toxicity [35] and better extraction efficiency than water [36]. Therefore, extraction methods based on aqueous ethanol are advantageous to extract PAC for applications in foods and nutraceuticals.

The objective of this study was to develop and optimize a PAC extraction method using food-grade ethanol as the extraction solvent to recover PAC from GS and grape seed powder (GSP). Aqueous ethanol has been used in both conventional [37] and advanced PAC extraction methods, including supercritical fluid extractions [34]. Extraction conditions such as temperature, together with the % ethanol, can significantly affect the recovery of PAC [38]. Therefore, the PAC extraction method for GS and GSP was optimized by central composite design (CCD) of response surface method (RSM) for three initial extraction parameters: % ethanol, extraction time, and solvent: solid (s:s) ratio. GS and GSP were originated from commercial grape varieties of *Vitis vinifera*, *V. labrusca*, and hybrids of native American varieties with *V. vinifera*. GS and GSP from a mixture of grape varieties were used with the intention of generalizing the new PAC extraction method for GS and GSP from different grape varieties. Ultrasonication and heat were later introduced to increase the efficiency of the extraction process. The extracted GS-PAC were characterized by high-performance liquid chromatography (HPLC), and retention of biological activity was tested in a palmitic acid-induced steatosis cell model. We further analyzed the PAC content of different grape varieties before and after fermentation in wine processing to determine the suitability of wine by-products as a source of PAC extraction. Identification of suitable PAC extraction sources and the development of food compatible extraction methods can benefit the development of functional foods and nutraceuticals enriched with PAC.

## 2. Results

### 2.1. Response Surface Models

A CCD of response surface modeling with 20 runs was used to optimize the PAC extraction from GS and GSP using aqueous ethanol as the extraction solvent. Initially, three extraction parameters: % ethanol, extraction time (h), and s:s ratio (*v:w*) were optimized. PAC yields extractable under combinations of low-axial, low, center, high, and high-axial levels of these parameters were determined by the methylcellulose (MC) precipitable tannin assay (Table 1). The experimental PAC yields were fitted to a second-order polynomial response surface model, and the significance of the model components was tested by analysis of variance (ANOVA) (Table 2). The response surface models were excellent in describing the variation of PAC yields with the three extraction parameters. Both GS and GSP models were non-significant (*p* > 0.05) for lack-of-fit (0.299 and 0.736, respectively). The adjusted *R^2^* for GS and GSP models were 97.38% and 99.89%, respectively.

A second optimization was performed by introducing heat and ultrasonication to reduce the extraction time and volume of 47% aqueous ethanol. Only the GS was selected for the second optimization as optimum conditions and predicted yields were almost similar for GS and GSP. Similar to the first optimization approach, PAC yields extractable under different combinations of extraction parameters; temperature (°C), s:s ratio (*v:w*), and sonication time (min) were determined (Table 3). A second-order polynomial model well described the variation of PAC yields by the extraction parameters. The lack-of-fit (0.182) of the model was not significant, and the adjusted *R^2^* was 99.08% (Table 4).

### 2.2. Optimization of the Aqueous Ethanol-Based PAC Extraction Conditions

#### 2.2.1. First Extraction Approach

**Figure 1:**
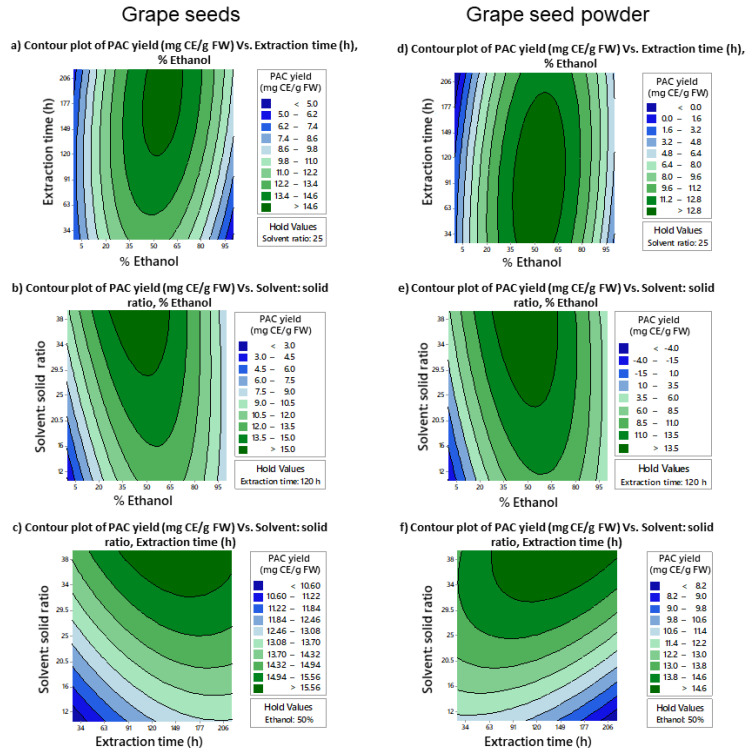
Contour plots of PAC yield variation (mg CE/g FW) under different extraction conditions and combinations of optimized parameters; % ethanol, solvent: solid ratio (*v:w*), and extraction time (h). Contour plots (**a**,**d**) describe the variation of PAC yields from grape seeds and grape seed powder, respectively, when % ethanol and extraction time are changed while holding the solvent: solid ratio at 25 (*v:w*). Contour plots (**b**,**e**) describe the variation of PAC yields under different combinations of % ethanol and solvent: solid ratio for grape seeds and grape seed powder, respectively, while holding extraction time at 120 h. Similarly, contour plots (**c**,**f**) describe the variation of PAC yields under different extraction times and solvent: solid ratios for grape seeds and grape seed powder, respectively, when % ethanol is held at 50%. mg CE/g FW, mg catechin equivalence/g fresh weight; PAC, proanthocyanidins; *v:w*, volume: weight.

**Figure 2:**
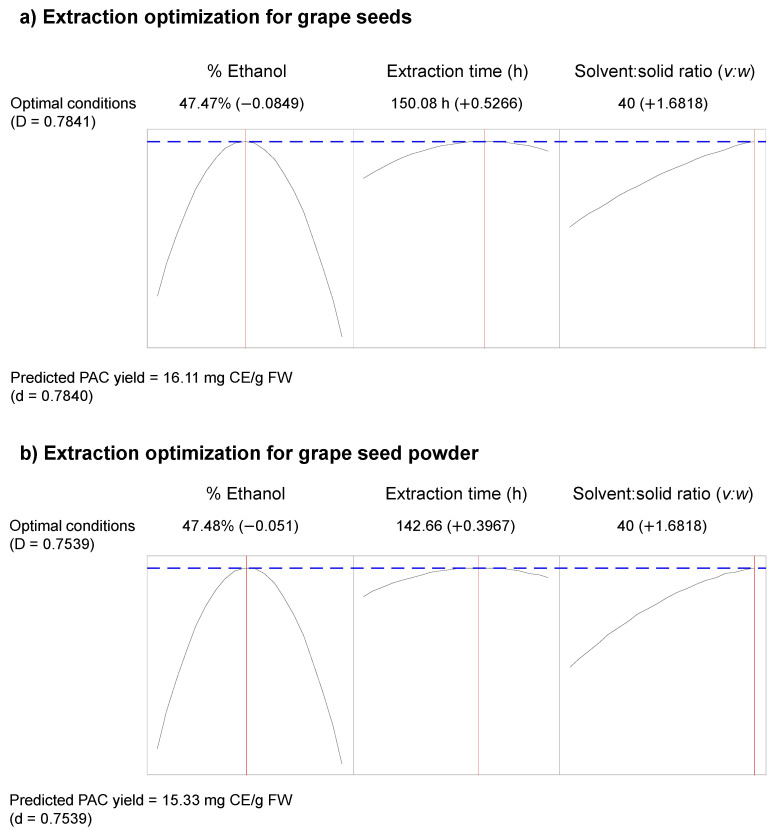
Optimization plots to predict maximum PAC yields and determine the optimum conditions to extract PAC from grape seeds (**a**) and grape seed powder (**b**). The optimization plots for grape seeds (**a**) predicted 47.47% ethanol and 150.08 h of extraction time as optimum conditions to extract PAC from grape seeds. The optimum solvent: solid ratio lies beyond the highest solvent: solid ratio (40 *v:w*) used in the experiment. At extraction conditions of 47.47% ethanol, 150.08 h of extraction time, and solvent: solid ratio of 40 (*v:w*), the predicted PAC yield for grape seeds is 16.11 mg CE/g FW. Similarly, the optimization plots for grape seed powder (**b**) predicted 47.48% ethanol and 142.66 h of extraction time as optimum conditions to extract PAC from grape seed powder. The optimum solvent: solid ratio lies beyond the highest solvent: solid ratio (40 *v:w*) used in the experiment. At extraction conditions of 47.48% ethanol, 142.66 h of extraction time, and solvent: solid ratio of 40 (*v:w*) the predicted PAC yield for grape seed powder is 15.33 mg CE/g FW. mg CE/g FW, mg catechin equivalence/g fresh weight; PAC, proanthocyanidins; *v:w*, volume: weight.

Contour plots (Figure 1 and Figure 2) were created to study the influence of selected extraction parameters on the PAC yield. In both GS and GSP extraction optimizations, extraction parameters showed a significant influence on the extractable PAC yield. Higher quantities of PAC were extractable from GS (>14.6 mg catechin equivalence/g fresh weight [mg CE/g FW]) and GSP (>12.8 mg CE/g FW) when the % ethanol was around 50%. Interestingly, PAC yield significantly dropped at higher % ethanol (Figure 1a,b,d,e). The PAC extraction time was slightly shorter for GSP compared to the GS (Figure 1a,d). The PAC yield continued to increase with the s:s ratio, suggesting the need to use higher volumes of aqueous ethanol in future extraction attempts (Figure 1b,c,e,f).

The extraction conditions were optimized by creating optimization plots with a focus to maximize the PAC yield (Figure 2). Optimization of the extraction process for GS predicted a maximum PAC yield of 16.1 mg CE/g FW (Figure 2a). Optimum extraction conditions were 47% of ethanol, 150 h of extraction time, and an s:s ratio of 40 (*v:w*). Optimum extraction conditions for GSP were closely similar to the GS. A maximum PAC yield of 15.3 mg CE/g FW was predicted for GSP when the extraction conditions were tuned to 47% of ethanol, 142.6 h of extraction time, and an s:s ratio of 40:1 (*v:w*) (Figure 2b). The contour plots and the optimization plots suggest an s:s ratio over 40:1 (*v:w*) to achieve the maximum yield of PAC. Thus, further incrementation of the s:s ratio may increase the extractable PAC yield, and the real optimum s:s ratio may be higher than 40:1 (*v:w*).

#### 2.2.2. Second Extraction Approach

**Figure 3:**
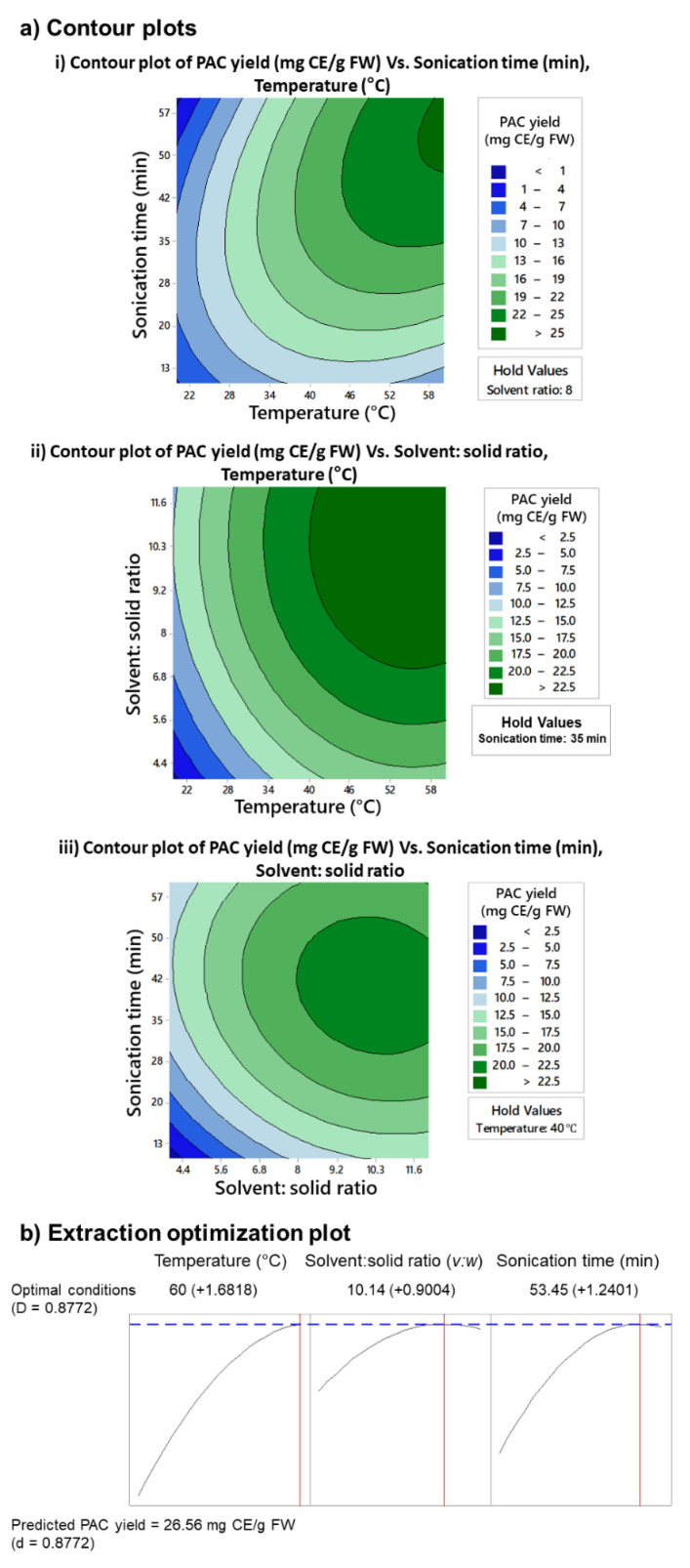
Contour plots (**a**) of PAC yield variation (mg CE/g FW) under different extraction conditions and combinations of optimized parameters: extraction temperature (°C), sonication time (min), and solvent: solid ratio (*v:w*); and optimization plots (**b**) to determine optimum PAC extraction conditions. Contour plot (**ai**) depicts the variation of PAC yield from grape seeds under different extraction temperatures and sonication time while holding solvent: solid ratio at 8 (*v:w*). Contour plot (**aii**) depicts the variation of PAC yields from grape seeds under different extraction temperatures and solvent: solid ratios while holding the sonication time at 35 min. Similarly, contour plot (**aiii**) describes the variation of PAC yields under different solvent: solid ratios and sonication times when extraction temperature is held at 40 °C. The optimization plots (**b**) predicted the optimum PAC extraction conditions to be 10.14 (*v:w*) solvent: solid ratio and 53.45 min sonication time. The optimum extraction temperature lies beyond the highest temperature (60 °C) tested in the study. At extraction conditions of 10.14 (*v:w*) solvent: solid ratio, 53.45 min sonication time, and extraction temperature of 60 °C, the predicted PAC yield from grape seeds was 26.56 mg CE/g FW. Ethanol at 47% was used to extract PAC as determined by first optimization approach. mg CE/g FW, mg catechin equivalence/g fresh weight; PAC, proanthocyanidins; *v:w*, volume: weight.

The initial extraction process was considered inefficient and resource intense. The extraction process required a large volume of aqueous ethanol (40 mL/g FW), and the extraction time was six days long. Therefore, a second extraction approach was attempted to reduce the extraction time and s:s ratio by introducing heat and ultrasonication to the extraction process. Previously optimized percent ethanol (47%) was used for the extraction process. Only GS was used in this optimization attempt, considering the similarity of predicted yields and optimum conditions for GS and GSP in the initial extraction processes. The tested extraction parameters, temperature, s:s ratio, and ultrasonication time significantly influenced the PAC yield of GS (Figure 3a). The predicted PAC yield (26.6 mg CE/g FW) was considerably higher compared to the first extraction approach. The PAC yield continued to increase with the increasing temperature, suggesting the possibility of using higher temperatures to increase the PAC yield (Figure 3(ai,aii)). The s:s ratio and the extraction time were considerably lower compared to the first extraction approach.

Optimization plots were created to identify the optimum extraction conditions to obtain the highest yield of PAC (Figure 3b). Optimum conditions to extract PAC from GS were a temperature of 60 °C, a s:s ratio of 10.14 (*v:w*), and an ultrasonication time of 53 min. The predictable PAC yield was 26.6 mg CE/g FW. The optimum temperature of 60 °C is the highest temperature tested in this extraction process. Considering the optimization plots and contour plots, more PAC could be extracted at temperatures higher than 60 °C. However, temperatures over 60 °C were not tested, considering the negative effect of high temperatures on the stability of PAC.

### 2.3. Evaluation of the Predicted PAC Yield and Comparison with Acetone-Based Extraction Method

Optimum conditions of the extraction parameters were tested to compare the PAC yield predicted in the second extraction approach. Under the optimum extraction conditions, 25.3 ± 1.26 mg CE/g FW of PAC, compared to the predicted value of 26.6 mg CE/g FW, was extracted from GS. The extracted PAC yield was not considerably different from that of the predicted PAC yield. The extracted PAC yield was significantly higher compared to the aqueous acetone-based extraction method. The PAC yield from the acetone-based extraction of GS was 9.15 ± 0.20 mg CE/g FW.

### 2.4. HPLC Analysis of the Sugar-Free Fraction of Extracted PAC

**Figure 4:**
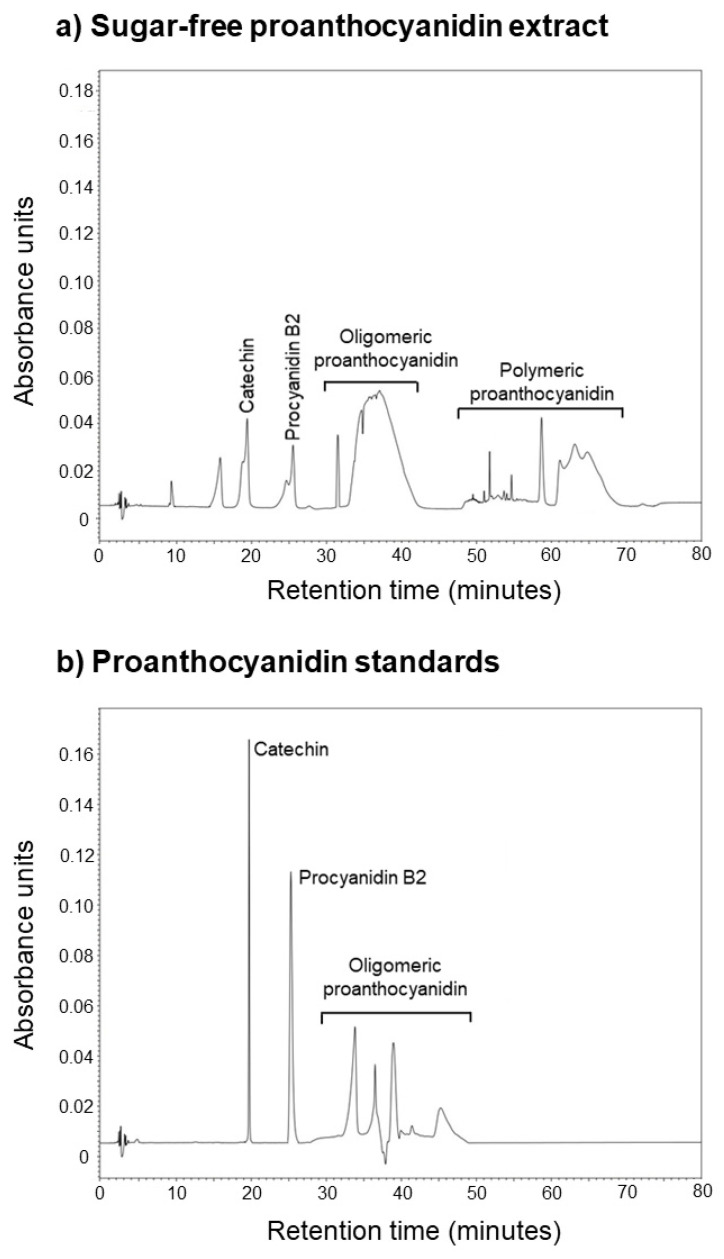
HPLC chromatogram of the sugar-free fraction of grape seed PAC (**a**); and chromatogram of catechin, procyanidin B2, and oligomeric grape seed PAC standard (**b**). PAC, proanthocyanidins.

The sugar-free fraction of the GS-PAC extracted by the optimized method was analyzed by HPLC to determine the monomeric, oligomeric, and polymeric composition. The HPLC chromatogram of sugar-free GS-PAC was compared with catechin (monomeric PAC), procyanidin B2 (dimeric PAC), and a GS-PAC oligomeric standard (Figure 4). The sugar-free fraction of GS-PAC was comprised of peaks for catechin (20 min), procyanidin B2 (26 min), and oligomeric PAC (33–43 min). Several peaks from 48–68 min were detected that might be attributed to polymeric PAC. The optimized aqueous ethanol-based PAC extraction method is suitable for extracting GS-PAC ranging from monomers to polymers.

### 2.5. Biological Activity of GS-PAC Extracted by the Optimized Method in Palmitic Acid-Induced Steatosis Model of AML12 Cells

**Figure 5:**
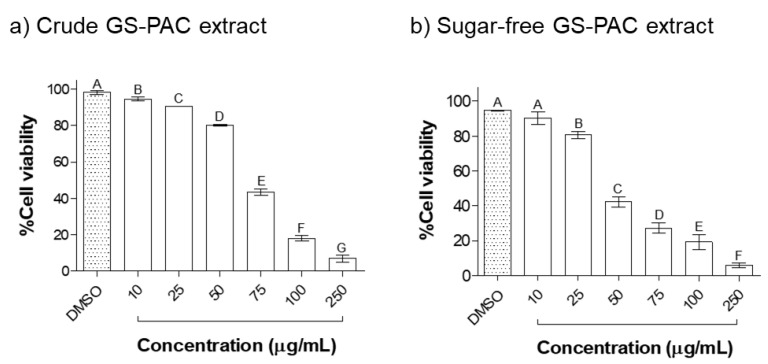
Toxicity of crude grape seed PAC (**a**) and sugar-free grape seed PAC (**b**) extracts on AML12 mouse liver cells. AML12 cells were treated with different concentrations (10–250 µg/mL) of crude and sugar-free extracts of grape seed PAC for 24 h. Cell viability was measured by MTS assay and expressed as % cell viabilities in comparison to the negative control. Results were given as mean ± *SD* of three independent experiments, and means that do not share a similar letter are significantly different (*p* ≤ 0.01). DMSO, dimethyl sulfoxide; PAC, proanthocyanidins.

Initially, non-toxic concentrations of crude and sugar-free GS-PAC extracts were determined by the MTS cell viability assay (Figure 5). The crude GS-PAC extract showed a considerable reduction in cell viability (<75%) at concentrations over 50 µg/mL. The sugar-free GS-PAC extract depicted higher toxicity in AML12 cells compared to the crude extract. Concentrations over 25 µg/mL of sugar-free GS-PAC extract reduced the % cell viability below 50%. Based on these results, 10, 25, and 50 µg/mL concentrations of crude and sugar-free GS-PAC extracts were selected to determine biological activity.

**Figure 6:**
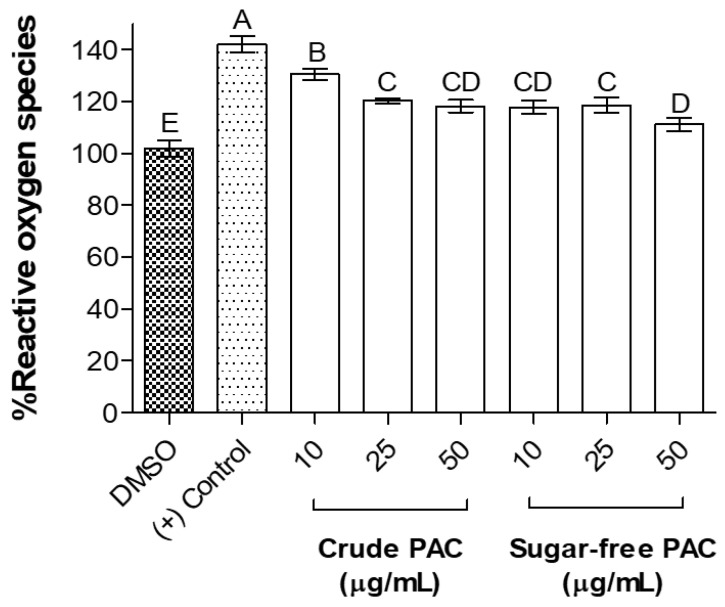
Potential of crude and sugar-free grape seed PAC extracts to ameliorate palmitic-acid-induced ROS in AML12 mouse liver cells. AML12 cells were pretreated with 10, 25, and 50 µg/mL concentrations of crude and sugar-free GS-PAC extracts for 24 h and exposed to palmitic acid (200 µM) for 24 h to induce ROS production. Cellular ROS production was measured by DCFDA cellular ROS detection assay and expressed as % ROS compared to negative control. Results were given as the mean ± *SD* of three independent experiments, and means that do not share a similar letter are significantly different (*p* ≤ 0.01). DMSO, dimethyl sulfoxide; PAC, proanthocyanidins.

Palmitic-acid-induced AML12 cells were used to assess the extracts for the reduction of cellular reactive oxygen species (ROS) generation and cellular lipid accumulation, which mimic steatosis. The ROS generation in AML12 cells was measured by the 2′,7′-dichlorofluorescein diacetate (DCFDA) cellular ROS detection assay (Figure 6). Exposure to palmitic acid (200 µM) alone (the experimental model) significantly increased the cellular ROS generation in AML12 cells. Both crude and sugar-free GS-PAC extracts significantly reduced the palmitic-acid-induced ROS generation by 10–20%. In crude GS-PAC-treated AML12 cells, reduction of ROS generation was dose-dependent. Interestingly, 25 and 50 µg/mL concentrations were equally effective in the reduction of cellular ROS generation. The 10 and 25 µg/mL concentrations of sugar-free GS-PAC were similarly effective in reducing cellular ROS generation (by 20%). The highest ROS suppression was observed for 50 µg/mL concentration of the sugar-free GS-PAC extract. However, this additional ROS reduction may be attributed to the loss of viable cells, as 50 µg/mL of sugar-free GS-PAC was toxic to AML12 cells.

**Figure 7:**
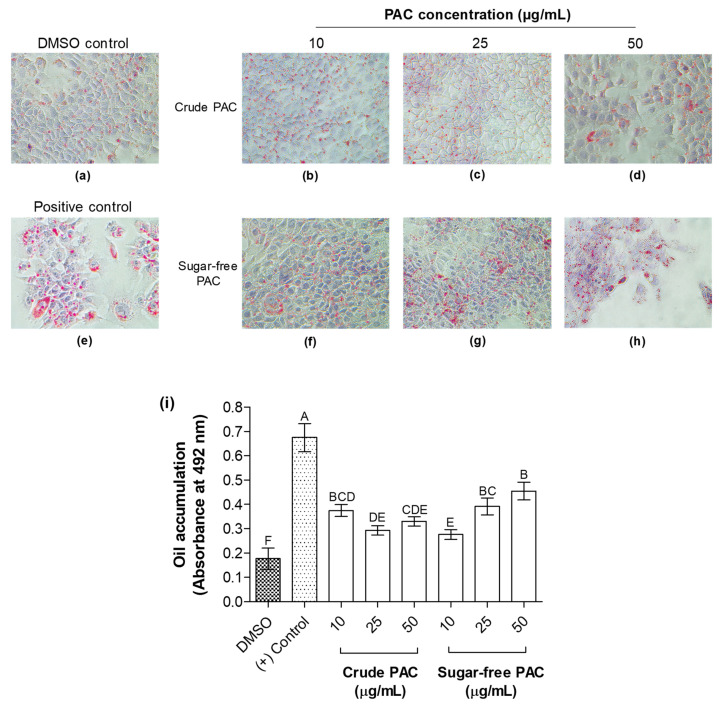
The potential of crude and sugar-free grape seed PAC extracts to reduce palmitic-acid-induced steatosis in AML 12 cells. AML12 cells were pretreated with 10, 25, and 50 µg/mL concentrations of crude grape seed PAC (**b**–**d**) and sugar-free grape seed PAC (**f**–**h**) extracts for 24 h and exposed to palmitic acid (200 µM) for 24 h to induce cellular lipid accumulation. Cells were stained with Oil Red O stain and visualized under a microscope (×100) to compare cellular lipid accumulation with vehicle (DMSO) control (**a**) and positive control (**e**). After microscopic visualization, Oil Red O stain retained in cells was extracted using 100% isopropanol. Oil Red O stain concentrations in isopropanol extracts were compared by measuring the absorbance at 492 nm as an indirect measurement of cellular lipid accumulation (**i**). Results were given as mean ± *SD* of three independent experiments, and means that do not share a similar letter are significantly different (*p* ≤ 0.01). DMSO, dimethyl sulfoxide; PAC, proanthocyanidins.

Cellular lipid accumulation in AML12 cells was visualized and measured by Oil Red O (ORO) staining of the cells (Figure 7). Both crude and sugar-free GS-PAC significantly reduced the palmitic-acid-induced lipid accumulation in cells. The reductions of lipid accumulation by different concentrations of crude GS-PAC extract were similar. The highest reduction of cellular lipid accumulation was observed for 10 µg/mL concentration of sugar-free GS-PAC extract. Interestingly, the 10 µg/mL concentration of sugar-free GS-PAC extract significantly reduced the cellular lipid accumulation greater than the 25 and 50 µg/mL concentrations did. Moreover, cellular lipid reduction by the 10 µg/mL concentration of sugar-free GS-PAC extract was statistically similar to that of the 25 and 50 µg/mL concentrations of crude GS-PAC extract. Both GS-PAC extracts depicted promising abilities to suppress palmitic-acid-induced steatosis in AML12 cells as measured by cellular ROS reduction and cellular lipid accumulation.

### 2.6. PAC Retention in Grape Mashes after Fermentation

**Figure 8:**
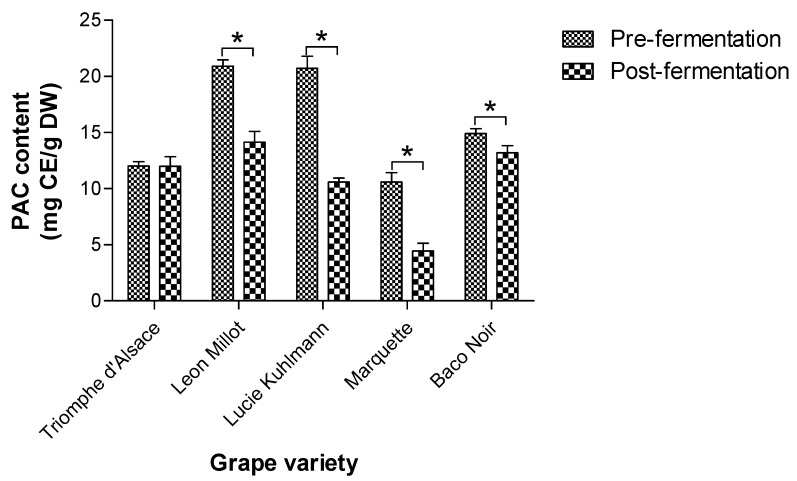
The PAC content of grape mashes from five different grape varieties before and after wine fermentation. PAC was extracted from grape mashes pre- and post-fermentation by the optimized method of PAC extraction (47% aqueous ethanol, 60 °C temperature, 10:1 solvent: solid ratio, and 53.45 min sonication time). PAC content of the extracts was measured by the methylcellulose precipitable tannin assay. * Means are significantly different at *p* < 0.01 level. mg CE/g DW, mg catechin equivalence/g dry weight; PAC, proanthocyanidins.

The PAC contents in grape mash from five common grape varieties (Triomphe d’Alsace, Leon Millot, Lucie Kuhlmann, Marquette, and Baco Noir) were quantified before and after fermentation for wine production (Figure 8). Initial PAC content (before fermentation) was high in the Leon Millot (20.9 mg CE/g dry weight [DW]) and Lucie Kuhlmann (20.7 mg CE/g DW) grape varieties compared to the other three grape varieties; Triomphe d’Alsace, Marquette, and Baco Noir. All the tested grape varieties, except for the Triomphe d’Alsace, showed a significant reduction in PAC content after the fermentation process. The Lucie Kuhlmann grape variety depicted the highest reduction in PAC content after the fermentation (20.7 to 10.6 mg CE/g DW). The PAC contents of the Leon Millet (20.9 to 14.1 mg CE/g DW) and Marquette (10.6 to 4.45 mg CE/g DW) grape varieties were also considerably lowered by the fermentation process. The Leon Millet (even after a significant PAC drop) and the Baco Noir grape varieties retained the highest quantities of PAC after the fermentation process (14.1 and 13.2 mg CE/g DW, respectively). Thus, grape by-products of wine production are a suitable source for PAC extraction, and it is important to consider the grape variety when using grape by-products for PAC extraction.

## 3. Discussion

The interest in utilizing PAC as functional food ingredients and nutraceuticals has continued to grow over time. The conventional PAC extraction methods require organic solvents such as ethyl acetate and acetone, which may leave harmful solvent residues along with extracted PAC. as well creating environmental impacts. Therefore, extraction methods solely based on water (e.g., subcritical water extraction) or aqueous food-grade ethanol are favorable in extracting PAC for food and nutraceutical applications [35]. However, extraction of PAC with only water requires higher extraction temperatures [36,39], which cause the degradation of PAC [40,41]. The antioxidant activity of GS-PAC declines at temperatures over 50 °C [40,41]. Moreover, the stability and yield of PAC by grape pomace are considerably reduced at temperatures over 60 °C [42]. Large quantities of GS have been generated annually as underutilized by-products of grape processing. GS accounts for around 13% of the grape’s weight and is rich in phenolic compounds (around 7%), including PAC [3]. Thus, GS is a viable source for PAC extraction on a large scale. The development of an aqueous ethanol-based PAC extraction method for GS will be beneficial to producing cost-effective, supplemented food and nutraceuticals enriched with PAC.

The optimum % ethanol predicted by the optimization plots for both GS and GSP was 47%. The extraction of PAC from GS is possible with water as the sole extraction solvent. The presence of ethanol can increase the rate of PAC extraction within the first six days of the extraction process [43]. Moreover, the yield of PAC increases with the % ethanol (0–15%), even though the extraction rates become identical after the sixth day of the extraction process [43]. The optimum % ethanol seems to rely on the plant source used to extract PAC. A much higher % ethanol (93.7%) is recommended as the optimum condition to recover PAC from Chinese wild rice [44]. The extraction times required for GS and GSP were closely similar (150 h Vs. 143 h). Also, the predictable PAC yields were not different between GS and GSP (16.1 mg CE/g FW Vs. 15.3 mg CE/g FW). Therefore, the grinding of grape seeds to create GSP is not required to increase the PAC extraction efficiency with aqueous ethanol. The optimum s:s ratio could not be determined for GS and GSP in the first optimization approach, as the aqueous ethanol requirement was over the maximum s:s ratio (40:1 *v:w*) used. This is obvious from the previous PAC extraction optimization studies. A s:s ratio of 50:1 is recommended as the optimum condition for maximizing the yield of PAC from Chinese wild rice when aqueous ethanol is used with ultrasound-assisted extraction [44]. Similarly, a s:s ratio of 58:1 (*v:w*) is reported for grape seeds extracted with methanol: acetone: water: acetic acid (30:42:7.5:0.5, *v:v:v:v*) in an ultrasonication-assisted solvent extraction process [45].

A second optimization approach was designed to increase PAC extraction efficiency and reduce the aqueous ethanol requirement. Heat and ultrasonication were introduced into the extraction process. Only GS was used, considering the similarity of predicted yields and optimum conditions between GS and GSP. The percentage of ethanol was fixed at 47% based on the knowledge from the first optimization approach. The introduction of heat and ultrasonication significantly reduced the extraction time (sonication time of 53 min) and aqueous ethanol requirement (10.14:1 *v:w*) while considerably increasing the PAC yield (16.1 mg CE/g FW Vs. 26.6 predicted mg CE/g FW). The optimum temperature for this ultrasonication-assisted extraction process could not be determined as the optimum condition was above the maximum extraction temperature (60 °C) used. However, higher extraction temperatures were not tested in this study as the stability and biological activity of PAC starts to drop above 50–60 °C [40,41,42]. The PAC yield may continue to increase with temperatures even higher than 100 °C. In a previous study, the PAC yield from GS continued to increase with temperature and maximized at 120 °C in a 70% aqueous acetone-based accelerated high-pressure extraction process [46]. The introduction of ultrasonication into the extraction process can significantly reduce the extraction time. Ultrasonication can disrupt the plant cell wall and improve the permeation of extraction solvent and target compounds through cell membranes [47].

The extraction solvent has a significant impact on the extractable yield of PAC [48]. The optimized aqueous ethanol-based ultrasonication-assisted PAC extraction method generated a significantly high PAC yield compared to the aqueous acetone-based extraction method (25.3 mg CE/g FW Vs. 9.1 mg CE/g FW). Several previous studies contradict this comparison and declare the superiority of acetone-based extractions over aqueous ethanol [46,48,49]. However, a study on the extraction of bioactive compounds from macadamia skin waste suggests that aqueous ethanol (50%) can extract more PAC compared to aqueous acetone (50%) [50]. The extractable PAC yield greatly varies depending on the extraction conditions. A higher yield of PAC may be possible to obtain from the acetone-based extraction method if the s:s ratio or extraction temperature is further increased. However, under the tested extraction conditions, the aqueous ethanol-based extraction method is superior to the acetone-based method. Therefore, the developed ethanol-based extraction method can be recommended for efficient scale-up recovery of PAC from GS by the industry.

The HPLC analysis of sugar-free GS-PAC established the ability of the optimized method to extract PAC, ranging from monomers to polymers. Sica et al. (2018) has observed a similar chromatogram for a GS extract by using a very sophisticated ultra-high performance liquid chromatography-charged aerosol detector (UHPLC-CAD) instrument [51]. A broad oligomeric PAC peak was detected by the CAD detector (similar to the peak between 33–43 min in Figure 4), and further analysis revealed this peak represents polymerized tannins with six or more monomeric catechin units [51]. A number of other studies have reported similar chromatograms in approaches to characterize PAC-rich GS extracts [52,53,54]. However, many of these chromatograms do not report a second broad PAC peak similar to the peak observed between 60–68 min (Figure 4) in this study. The second broad peak can be a mixture of highly polymerized PAC, and further studies can be recommended for its resolution.

Since the aqueous ethanol-based PAC extraction method was developed with the intention of generating PAC suitable for applications in food and nutraceuticals, it is important to establish the retaining of the biological activity of PAC after the extraction process. A cell model of steatosis using palmitic-acid-induced AML12 cells was used to test the biological activity of extracted crude and sugar-free GS-PAC. Initial cell viability assays revealed the dose-dependent toxicity of extracted GS-PAC on AML12 cells. The toxicity of PAC in cells has previously been established by several studies, especially for cancer cells [55,56,57]. PAC can induce cellular ROS generation and cause apoptosis in a dose-dependent manner. A considerable loss of cell viability (<80%) was observed in cells treated with low concentrations of PAC (>20 µg/mL) from multiple plant sources [55,57]. Higher cytotoxicity was observed for the purified, sugar-free GS-PAC extract compared to the crude GS-PAC extract. The high toxicity of the sugar-free GS-PAC extract can be attributed to the high PAC concentration in the purified extract after flash chromatography.

GS-PAC extracted by the optimized method was able to ameliorate steatosis markers in AML12 cells incubated with palmitic acid. Cellular ROS generation was measured in the AML12 cells as steatosis is associated with increased generation of ROS in hepatocytes [58]. PAC can reduce cellular ROS generation through multiple mechanisms. PAC is a natural antioxidant; therefore, it can neutralize cellular ROS by direct scavenging. PAC can also modulate multiple cell-signaling pathways to alleviate cellular ROS generation. Activation of nuclear factor erythroid 2-related factor 2 (Nrf2) [59], suppression of mitogen-activated protein kinase (MAPK), nuclear factor-kappa B (NF-*κ*B), and phosphoinositide 3-kinase (PI3K)/Akt pathways have been identified as PAC-mediated interventions to reduce cellular oxidative stress [60]. Procyanidin B2 was found in the GS-PAC extracted by the optimized method. Procyanidin B2 helps to maintain cellular redox homeostasis by protecting mitochondrial membrane potential and improving the activity of cellular antioxidant enzymes superoxide dismutase (SOD), glutathione peroxidase (GPx), and catalase (CAT) in hepatocytes [61]. Similar to ROS generation, lipid accumulation was also measured as a marker of steatosis in AML12 cells. Extracted GS-PAC was able to significantly reduce palmitic-acid-induced lipid accumulation in AML12 cells. This reduction of cellular lipid accumulation can be explained by the effects of PAC on cell signaling pathways regulating lipid degradation and lipogenesis. Procyanidin B2 can promote lysosomal-mediated lipid degradation by upregulating the expression of Lamp1, Mcoln, and Uvrag genes through modulating transcription factor EB (TFEB). Also, PAC B2 restricts lipogenesis by the downregulation of peroxisome proliferator-activated receptor γ (PPARγ), CCAAT/enhancer-binding protein α (C/EBPα), and sterol regulatory element-binding protein-1 (SREBP-1) genes [61]. A similar result has been reported by Yogalakshmi et al. (2013) when GS-PAC was supplemented to mice fed with a high-fat-high-fructose diet to induce steatosis [62]. Therefore, interventions of GS-PAC can alleviate steatosis by inhibiting fatty acid synthesis in mice [62].

We tested the suitability of grape by-products from wine production for the extraction of PAC using the optimized method. The PAC content of grape mashes from five grape varieties was quantified before and after the fermentation process. All the tested grape varieties except for Triomphe d’Alsace depicted a significant reduction in the PAC content after fermentation. The loss of PAC from the initial grape mash during fermentation is evident by the presence of PAC in wine [63,64]. It is the polymeric fraction of PAC (degree of polymerization >12–15) abundant in both red and white wines [63]. Thus, the reduction of PAC from grape mashes after fermentation may result from the loss of polymeric PAC. The grape mash consists of grape skins, flesh, stems, and seeds. About half of the monomeric and oligomeric PAC in GS leaches into the wine during fermentation. However, GS does not contribute to the polymeric PAC in wine. The polymeric PAC in wine mainly originates from the grape skins and stems [65]. Moreover, red wine contains more PAC compared to white wine [63]. Therefore, the selection of grape by-products from white wine production can be useful for extracting a higher yield of PAC. However, it is also important to consider the grape variety, as PAC content before and after fermentation greatly varies depending on the grape variety.

## 4. Materials and Methods

### 4.1. Materials and Chemicals

The GS and GSP used in this study were provided by the Royal Grapeseed, Milton, NY, USA. Both the GS and GSP came from a mixture of commercial grape varieties; *Vitis vinifera*, *V. labrusca*, and hybrids of native American species with *V. vinifera*. Grape mash before and after fermentation in wine processing was provided by the Jost Vineyards, Malagash, NS, Canada. Grape mashes from five different grape varieties, Triomphe d’Alsace, Leon Millot, Lucie Kuhlmann, Marquette, and Baco Noir, were tested in this study. Anhydrous ethanol (P016EAAN) for PAC extraction was purchased from Commercial Alcohols, Brampton, ON, Canada. Acetone (A18) for the conventional extraction of PAC and isopropanol (BP2618) were purchased from Thermo Fisher Scientific, Fair Lawn, NJ, USA. Dulbecco’s Modified Eagle Medium/Nutrient Mixture F-12 (DMEM:F-12) cell culture medium (30-2006^TM^) was purchased from American Type Culture Collection (ATCC^®^), Manassas, VA, USA. Phenol-red-free DMEM:F-12 culture medium (Gibco^TM^ 21041025) was purchased from Fisher Scientific, Saint-Laurent, QC, Canada. Bovine serum albumin (BSA), catechin (C1251), DCFDA (D6883), dexamethasone, dimethyl sulfoxide (DMSO), fetal bovine serum (FBS), insulin-transferrin-selenium (ITS) liquid media supplement (I3146), MC cP 1500 (M0387), ORO stain (O0625), paraformaldehyde, palmitic acid (P5585), penicillin-streptomycin, phenazine methosulphate (PMS), phosphate-buffered saline (PBS), and procyanidin B2 (42157) were purchased from MilliporeSigma, Oakville, ON, Canada. Oligomeric PAC from grape seeds (1298219) was purchased from USP, Rockville, MD, USA, to use as a standard for HPLC analysis. CellTiter 96^®^ aqueous MTS reagent powder (G1111) was purchased from Promega Corporation, Madison, WI, USA.

### 4.2. Cell Line and Culture Conditions

AML12 normal mouse liver cells were used in experiments for evaluating the biological activity of extracted GS-PAC by means of their potential to reduce cellular ROS generation and lipid accumulation. AML12 cells (CRL-2254^TM^) were purchased from ATCC^®^, Manassas, VA, USA. DMEM: F-12 cell culture medium was supplemented with FBS (10%), dexamethasone (40 ng/mL), penicillin-streptomycin (50 U/mL), and ITS liquid media supplement (10 µg/mL, 5.5 µg/mL, and 5 ng/mL, respectively) to prepare the complete growth medium. AML12 cells were cultured at 37 °C in a humidified incubator with a 5% CO_2_ atmosphere. The cells were supplied with fresh, complete growth media for every 2 to 3 days and sub-cultured at 80% confluence.

### 4.3. Experimental Design

The CCD of RSM was used to determine the optimal conditions of PAC extraction using aqueous ethanol as the extraction solvent. The CCD consisted of 20 runs of combinations of three optimization parameters at the low-axial, low, center, high, and high-axial levels. Initially, % ethanol in water, solvent: solid (s:s) ratio (*v:w*), and extraction time (h) were optimized (Table 5). The initial optimization approach resulted in a significantly long extraction time and required a large volume of extraction solvent. To reduce the extraction time and extraction solvent volume, ultrasonication combined with heating was introduced into the extraction process. A second optimization was performed using a CCD of twenty runs with three optimization parameters; temperature (°C), s:s ratio (*v:w*), and sonication time (min), similar to the initial optimization approach (Table 5). The CCD was generated and analyzed using Minitab 18 (version 18.1) statistical software to determine the optimal conditions for the recovery of PAC from GS and GSP using aqueous ethanol as the extraction solvent.

### 4.4. Extraction of PAC by Aqueous Ethanol

Prior to the extraction process, GS were cleaned by removing any debris and damaged seeds. GS or GSP (1.0 g) were weighed into 50 mL plastic tubes. Anhydrous ethanol was mixed with deionized (DI) water to prepare the ethanol-based extraction solvent at different levels (0, 20.24, 50, 79.76, and 100 %), agreeing with the CCD. GS and GSP were mixed with the ethanol extraction solvent (10, 16.07, 25, 33.92, or 40 mL) in 50 mL plastic tubes. The extraction process was performed for different periods (24, 62.86, 120, 177.14, or 216 h) while slowly shaking (50 rpm) the tubes at 20 °C. The optimum conditions for the extraction process were statistically determined by the CCD of RSM.

A second PAC extraction was performed to reduce the extraction time and extraction solvent volume by introducing ultrasonication and heating into the extraction process. Only GS were used in this extraction process, as a large difference was not observed between GS and GSP for the PAC yields and optimum conditions of the tested parameters. Based on the first optimization approach, the ethanol level was set at 47% during this second optimization. Similar to the first extraction, GS (1.0 g) was weighed into 50 mL plastic tubes and mixed with different volumes of 47% ethanol in DI water (4, 5.62, 8, 10.38, or 12 mL). The extraction process was performed at different temperatures (20, 28.1, 40, 51.9, or 60 °C) while ultrasonicating for 10, 20.12, 35, 49.88, or 60 min. The optimum conditions for the extraction process were established by the CCD of RSM statistical method. The accuracy of the predicted optimized parameters was tested by performing the extraction process at the optimum values generated by the CCD analysis. The optimum conditions predicted by the CCD analysis were 47% ethanol, 10:1 s:s ratio (*v:w*), 53 min of sonication time, and extraction temperature of 60 °C. The accuracy of the predicted optimum values was evaluated by the MC precipitable tannin assay in three individual experiments.

### 4.5. Conventional Extraction of PAC

Conventional extraction of PAC was conducted as described by Rupasinghe et al. (2019) [66] with modifications to compare with the ethanol-based extraction method. Briefly, the extraction solvent was prepared by mixing acetone and formic acid with DI water (70: 0.1: 29.9 *v:v:v*). The original extraction s:s ratio was increased from 6:1 to 12:1 in order to match the optimum value of the aqueous ethanol-based extraction method. The extraction temperature was also increased to 60 °C. GS or GSP (1.0 g) mixed with the acetone-based extraction solvent (12 mL) were sonicated for 30 min × 3, with 10 min intervals in between to prevent the temperature from rising. This extraction process was individually performed three times.

### 4.6. Quantification of PAC in Extracts

PAC content in extracts was quantified by MC precipitable tannin assay modified to high throughput 96-well format [67]. All extracted samples were initially filtered through coarse porous (20–25 µm) filter papers. The filtrates (only 1 mL) were filtered again using 0.22 µm nylon filters. Standard catechin solutions (1–100 µg/mL) were prepared in 50% ethanol.

MC solution of 0.04% was prepared to quantify the PAC in filtered extracts. Initially, 0.4 g of MC was slowly dissolved in 300 mL of DI water at 80 °C while magnetic stirring to prevent clumping. After completely dissolving MC, 650 mL of ice-cold water (0–5 °C) was mixed with the hot MC solution. We continued stirring ice-cold water into the MC solution for 40 min and ultrasonicated it for 15 min. The final MC solution was marked up to 1 L in a volumetric flask.

To measure the PAC quantity, 100 µL of filtered extract or catechin standard was mixed with 300 µL of MC solution, 200 µL of saturated (NH_4_)_2_SO_4_ solution, and 400 µL of DI water in 1.5 mL Eppendorf tubes. The controls were prepared for each extracted sample and catechin standard by replacing the MC solution with an extra 300 µL of DI water. Eppendorf tubes were centrifuged at 10,000 × *g* for 5 min, and the supernatants were pipetted (200 µL/well) into a UV-transparent 96-well plate (Corning Incorporated, Kennebunk, ME, USA) in triplicate. The absorbance of the wells was measured at 280 nm (Infinite^®^ M200 PRO, Tecan Trading AG, Mannedorf, Switzerland), and the absorbance differences between extracts/catechin standards and respective controls were calculated. A catechin standard curve was created by plotting the absorbance difference against catechin concentration. PAC quantity in extracted samples was calculated using the catechin standard curve, and the results were expressed in mg CE/g.

### 4.7. Purification of Crude GS-PAC to Generate a Sugar-Free Fraction

Crude GS-PAC was purified by flash chromatography to generate a sugar-free PAC extract. The sugar-free fraction of GS-PAC was used for HPLC analysis and AML12 cell-based experiments to evaluate biological activity. Initially, crude PAC was extracted from GS under the optimized conditions (50 g in 500 mL of 47% ethanol in water). Part of the PAC extract (300 mL) was loaded into a glass flash chromatography column (6.5 × 45 cm, Sati International Scientific Inc., Dorval, QC, Canada) packed with 400 g of Sorbent beads (Sorbent SP207-05 Sepabeads resin brominated styrenic adsorbent, Sorbent Technologies, Atlanta, GA, USA) and eluted with DI water until Brix value reached below 0.1% (no sugars present in the elute). Then, PAC was eluted by using 80% acetone in water and concentrated by rotary evaporation at 35 °C until the PAC concentrate was free of acetone. The crude GS-PAC extract (200 mL) was also concentrated by rotary evaporation until the PAC concentrate was free of ethanol. Concentrated PAC samples were freeze-dried for 24 h and stored at −80 °C.

### 4.8. HPLC Analysis of PAC Extracted by the Ethanol-Based Optimized Method

The composition of the sugar-free GS-PAC extract was determined by HPLC analysis. Catechin (25 µg/mL), procyanidin B2 (25 µg/mL), and oligomeric PAC from grape seeds (50 µg/mL) were dissolved in 80% ethanol in water to prepare the standards for HPLC analysis. Sugar-free GS-PAC extract (200 µg/mL) was also dissolved in 80% ethanol in water to inject into HPLC. All samples were syringe-filtered through 0.22 µm filters before injecting into the HPLC. The HPLC system used for this analysis consisted of a Waters 2695 separation module coupled with a Waters 2996 photodiode array (PDA) detector (Waters, Milford, MA, USA) and equipped with an Xselect^®^ HSS C_18_ 5 µm column (4.6 × 150 mm, Waters, Milford, MA, USA). The HPLC separation was performed by using 0.1% formic acid in water (solvent A) and acetonitrile (solvent B) as the mobile phases at a flowrate of 0.7 mL/min. The HPLC run method was comprised of linear and isocratic gradients of mobile phases, where the proportion of acetonitrile (% solvent B) was altered over time; 5–15% from 0–45 min (linear), 15–80% from 45–50 min (linear), 80% from 50–53 min (isocratic), and 5% from 53–70 min (isocratic recalibration). HPLC sample injection volume was 10 µL, and PDA detection was set to 280 nm wavelength.

### 4.9. Evaluation of the Biological Activity of Extracted GS-PAC

The biological activity of extracted GS-PAC was evaluated by using the palmitic-acid-induced steatosis model of AML12 mouse liver cells. Palmitic acid was solubilized in 20% BSA in PBS as described in Zeng et al. (2020) [68] to produce a stock solution of 10 mM. AML12 cells were treated with different concentrations of palmitic acid (10–750 µM) for 24 h, and the development of ROS and the cellular accumulation of lipids were measured by DCFDA assay and ORO staining of cells, respectively (data not shown). Cell viability of AML12 cells under different concentrations of palmitic acid was determined by MTS cell viability assay (data not shown). A concentration of 200 µM of palmitic acid was selected for further experiments considering low toxicity to AML12 cells (> 90% cell viability) and potential to promote ROS generation (142% compared to negative control) and cellular lipid accumulation.

#### 4.9.1. Determination of Toxicity of GS-PAC in AML12 Cells

The toxicities of crude GS-PAC and purified sugar-free GS-PAC extracted by the optimized extraction method were evaluated by the MTS cell viability assay. AML12 cells were seeded in 96-well plates at a density of 10,000 cells/well and incubated overnight under normal culture conditions. Then, cells were treated with different concentrations of crude and sugar-free GS-PAC (1–1000 µg/mL) for 24 h. After treatment with PAC, 20 µL of MTS/PMS solution (MTS, 333 µg/mL and PMS, 25 µM of final concentration) was added into wells and incubated for 3 h at normal culture conditions. After incubation, absorbance for each well was measured at 490 nm using a microplate reader (Infinite^®^ M200 PRO, Tecan Trading AG, Mannedorf, Switzerland). The absorbance values between the PAC treatments and control were compared and expressed in percentages as an indirect measurement of cell viability.

#### 4.9.2. Evaluation of Extracted GS-PAC to Alleviate Palmitic Acid-Induced ROS Generation

The potential of GS-PAC obtained by optimized extraction method in mitigation of palmitic-acid-induced ROS generation in AML12 cells was studied by DCFDA assay. A 100 mM stock solution of DCFDA was prepared by dissolving in DMSO. AML12 cells were seeded in black 96-well plates for fluorescence-based assay at a density of 10,000 cells/well and incubated overnight under normal culture conditions. Then, cells were pre-treated with 10, 25, and 50 µg/mL concentrations of crude and sugar-free GS-PAC for 24 h. After treatment with PAC, cellular ROS generation was induced by exposing cells to 200 µM palmitic acid diluted in complete DMEM:F-12 culture medium for 24 h. A 5 µM working solution of DCFDA was prepared by diluting the stock solution in a complete DMEM:F-12 culture medium. AML 12 cells exposed to palmitic acid were gently washed with PBS, and 200 µL of DCFDA working solution was added to each well. After incubation for 30 min under normal culture conditions, cells were gently washed with PBS three times, and 200 µL of phenol red-free DMEM:F-12 culture medium was added to each well. The fluorescence of each well was measured at 485 nm (excitation)/535 nm (emission) using a microplate reader (Infinite^®^ M200 PRO, Tecan Trading AG, Mannedorf, Switzerland).

#### 4.9.3. Evaluation of Extracted GS-PAC to Alleviate Palmitic Acid-Induced Steatosis In Vitro

The ability of crude and sugar-free GS-PAC obtained by the optimized extraction method to alleviate palmitic-acid-induced steatosis in AML12 cells was studied by the ORO staining of cellular lipids. AML12 cells were seeded in 6-well plates at a density of 2.5 × 10^5^ cells/well and incubated overnight under normal culture conditions. Then, cells were treated with 10, 25, and 50 µg/mL concentrations of crude and sugar-free GS-PAC for 24 h and exposed to 200 µM palmitic acid diluted in complete DMEM:F-12 culture medium to induce cellular lipid accumulation. A stock solution of ORO stain was prepared by dissolving 60 mg of ORO powder in 20 mL of isopropanol. The stock solution was mixed with DI water (3:2 *v*/*v*) and syringe-filtered through 0.22 µM filters to create the ORO stain working solution. AML12 cells exposed to palmitic acid were gently washed with PBS twice and fixed by incubating with 4% paraformaldehyde solution for 45 min at room temperature (RT). After fixation, cells were gently washed twice with DI water and permeabilized by incubating with 60% isopropanol in DI water for 5 min at RT. Cellular lipid was stained with ORO by incubating cells with ORO working solution for 15 min at RT. After staining, cells were gently washed with DI water three times and incubated with hematoxylin nuclear stain for 1 min at RT. Excess hematoxylin was removed by gently washing the cells with DI water (3 times), and microscopic images (at × 100) of stained cells were observed while cells were immersed in DI water. Palmitic-acid-induced lipid accumulation in cells was indirectly measured by quantifying ORO stain retention in cells. To quantify cellular retention of ORO stain in AML12 cells, first, the plate wells were washed three times with 60% isopropanol in DI water. Then, the ORO stain in the cells was extracted by using 1 mL of 100% isopropanol and slowly rocking the 6-well plate for 5 min. The ORO stain concentrations in isopropanol extracts were expressed as absorbance values at 492 nm.

### 4.10. Extraction of PAC from Grape Mashes before and after Fermentation

PAC retention in grape mashes after fermentation for wine production was measured to determine the suitability of wine by-products as a source of PAC extraction. The mashes before fermentation consisted of grape skin, flesh, stems, and seeds. After the fermentation process, only grape seeds and skin were available as the by-products. Five different grape varieties, Triomphe d’Alsace, Leon Millot, Lucie Kuhlmann, Marquette, and Baco Noir, were tested in this study for the retention of PAC after fermentation. Grape mashes before and after fermentation of wine processing were freeze-dried for 48 h. Freeze-dried samples were ground into a fine powder, and PAC was extracted by the optimized PAC extraction method. Briefly, 1 g of powdered grape mash was mixed with 10 mL of 47% ethanol (in water) and ultrasonicated for 53 min at 60 °C. Extracted PAC quantity was measured by the MC precipitable tannin assay.

### 4.11. Statistical Analysis

Statistical analysis was conducted as previously described by Chen et al. (2020) [22]. Optimum conditions for extracting the maximum yield of PAC from GS and GSP were determined by CCD of RSM with 20 runs. Normal distribution and constant variance of the error terms were established by normal probability plots and residual versus fits plots. The independence was assumed by the randomization of run order. A second-order polynomial model was used to describe the variation of the response variable (PAC yield) by the independent variables (extraction parameters), and the regression coefficients were calculated.

Y = β_0_ + β_1_ X_1_ + β_2_ X_2_ + β_3_ X_3_ + β_1_^2^ X_1_^2^ + β_2_^2^ X_2_^2^ + β_3_^2^X_3_^2^ + β_1_ β_2_ X_1_ X_2_ + β_1_ β_3_ X_1_ X_3_ + β_2_ β_3_ X_2_ X_3_

Y is the PAC yield and β_0_ is the constant; β_1_, β_2_, and β_3_ are the regression coefficients of linear effect terms; β_1_^2^, β_2_^2^, and β_3_^2^ are the quadratic effect terms and β_1_ β_2_, β_1_ β_3_, and β_2_ β_3_ are the interaction effect terms; X_1_, X_2_, and X_3_ are the three extraction parameters tested.

The adequacy of the model was confirmed by the non-significant lack of fit (*p* > 0.05). The significance of each term (extraction parameter) was tested by analysis of variance (ANOVA), and contour plots were constructed. The optimum condition of each parameter to extract the maximum content of PAC was determined by the response optimizer. The mean PAC content of different PAC extracts was compared by the one-way ANOVA at 0.05 significance level. The differences in mean % cell viabilities, cellular ROS generation, cellular lipid accumulation, and PAC quantities in the grape mash before and after fermentation were also tested by the one-way ANOVA at a 0.05 significance level. The CCD generation and all statistical analyses were performed by Minitab^®^ statistical software (version 18.1).

## 5. Conclusions

The development of an aqueous ethanol-based PAC extraction method is beneficial for the supplemented food and nutraceutical industries. The optimum ethanol concentration for the extraction of PAC from GS and GSP is 47%. PAC yields and optimum extraction conditions are similar for GS and GSP. Therefore, grinding grape seeds into flour is not necessary for increasing the yield and extraction efficiency with aqueous ethanol. The extraction of PAC from GS and GSP using aqueous ethanol at room temperature is highly inefficient and requires a large volume of ethanol. The introduction of ultrasonication and heating into the extraction process can significantly reduce the extraction time and aqueous ethanol requirement while increasing the PAC yield. The optimized conditions to extract PAC from GS are 47% aqueous ethanol, 60 °C, 10.14:1 s:s ratio, and 53 min of sonication time. The developed aqueous ethanol-based extraction method can generate a higher yield of PAC from GS compared to the conventional acetone-based extraction method. HPLC analysis of sugar-free GS-PAC revealed that the optimized method could extract catechin, procyanidin B2, oligomeric, and polymeric PAC from GS. Both crude and sugar-free GS-PAC extracted by the optimized method depict toxicity in AML12 mouse hepatocytes at low concentrations (>25 µg/mL). Also, extracted GS-PAC could ameliorate palmitic-acid-induced steatosis in AML12 cells by reducing cellular ROS generation and lipid accumulation. The PAC content of grapes greatly varies depending on the variety. A significant reduction of PAC occurs in the grape mash during fermentation for wine production. However, grape by-products, even after fermentation, retain enough PAC to consider them as a source of PAC. The developed extraction method can be recommended for extracting PAC from GS and grape by-products from wine production without losing biological activity to use in supplemented food and nutraceutical products.

## Figures and Tables

**Table 1:** Central composite design (CCD) used in the first optimization approach and the proanthocyanidin yields under different combinations of extraction conditions.

				Proanthocyanidin Yield (mg CE/g FW)	
Run Order	Ethanol (%)	Extraction Time (h)	Solvent: Solid Ratio (*v:w*)	Grape Seeds	Grape Seed Powder
1	20.24 (−1)	62.86 (−1)	33.92 (+1)	13.17	11.19
2	79.76 (+1)	177.14 (+1)	16.07 (−1)	12.63	9.37
3	79.76 (+1)	62.86 (−1)	16.07 (−1)	10.58	9.97
4	50 (0)	120 (0)	25 (0)	14.84	13.60
5	100 (+1.68)	120 (0)	25 (0)	8.30	4.66
6	50 (0)	120 (0)	25 (0)	14.30	13.92
7	0 (−1.68)	120 (0)	25 (0)	6.81	1.58
8	79.76 (+1)	177.14 (+1)	33.92 (+1)	13.11	11.51
9	20.24 (−1)	177.14 (+1)	16.07 (−1)	9.99	4.84
10	50 (0)	120 (0)	25 (0)	14.65	13.69
11	20.24 (−1)	62.86 (−1)	16.07 (−1)	9.40	7.43
12	50 (0)	120 (0)	25 (0)	14.61	13.94
13	20.24 (−1)	177.14 (+1)	33.92 (+1)	12.56	10.22
14	79.76 (+1)	62.86 (−1)	33.92 (+1)	11.13	10.31
15	50 (0)	120 (0)	25 (0)	14.12	13.70
16	50 (0)	24 (−1.68)	25 (0)	12.41	13.36
17	50 (0)	120 (0)	25 (0)	15.10	13.78
18	50 (0)	216 (+1.68)	25 (0)	15.19	11.95
19	50 (0)	120 (0)	40 (+1.68)	16.40	15.20
20	50 (0)	120 (0)	10.00 (−1.68)	12.13	10.28

Low-axial (−1.68), low (−1), center (0), high (+1), and high-axial (+1.68) levels of the three extraction parameters are indicated in brackets. mg CE/g FW, mg catechin equivalence/g fresh weight.

**Table 2:** Analysis of variance (ANOVA) *p*-values and regression coefficients for the second-order response surface model in terms of the first optimization approach.

	Grape Seeds		Grape Seed Powder	
Source of Variance/Terms	*p*-Value	Regression Coefficient	*p*-Value	Regression Coefficient
Constant	0.000	14.607	0.000	13.7654
% Ethanol	0.010	0.354	0.000	0.9276
Extraction time	0.000	0.636	0.000	−0.3902
Solvent ratio	0.000	1.066	0.000	1.4569
% Ethanol × % Ethanol	0.000	−2.521	0.000	−3.7419
Extraction time × Extraction time	0.016	−0.312	0.000	−0.3704
Solvent ratio × Solvent ratio	0.201	−0.148	0.000	−0.342
% Ethanol × Extraction time	0.006	0.507	0.000	0.5214
% Ethanol × Solvent ratio	0.001	−0.663	0.000	−0.8329
Extraction time × Solvent ratio	0.301	−0.158	0.000	0.4285
Lack-of-fit	0.299		0.736	

Solvent ratio, solvent: solid ratio (*v:w*).

**Table 3:** Central composite design (CCD) used in the second optimization approach to reduce extraction time and solvent volume and to increase the proanthocyanidin yields from grape seeds.

Run Order	Temperature (°C)	Solvent: Solid Ratio (*v:w*)	Sonication Time (min)	Proanthocyanidin Yield (mg CE/g FW)
1	28.1 (−1)	5.62 (−1)	49.88 (+1)	8.39
2	51.9 (+1)	10.38 (+1)	20.12 (−1)	18.00
3	51.9 (+1)	5.62 (−1)	20.12 (−1)	12.45
4	40 (0)	8 (0)	35 (0)	19.68
5	60 (+1.68)	8 (0)	35 (0)	21.82
6	40 (0)	8 (0)	35 (0)	19.06
7	20 (−1.68)	8 (0)	35 (0)	7.77
8	51.9 (+1)	10.38 (+1)	49.88 (+1)	25.80
9	28.1 (−1)	10.38 (+1)	20.12 (−1)	13.61
10	40 (0)	8 (0)	35 (0)	18.98
11	28.1 (−1)	5.62 (−1)	20.12 (−1)	6.48
12	40 (0)	8 (0)	35 (0)	19.85
13	28.1 (−1)	10.38 (+1)	49.88 (+1)	12.32
14	51.9 (+1)	5.62 (−1)	49.88 (+1)	20.00
15	40 (0)	8 (0)	35 (0)	19.80
16	40 (0)	4 (−1.68)	35 (0)	11.35
17	40 (0)	8 (0)	35 (0)	19.80
18	40 (0)	12 (+1.68)	35 (0)	20.36
19	40 (0)	8 (0)	60 (+1.68)	17.58
20	40 (0)	8 (0)	10 (−1.68)	10.25

Low-axial (−1.68), low (-1), center (0), high (+1), and high-axial (+1.68) levels of the three extraction parameters are indicated in brackets. mg CE/g FW, mg catechin equivalence/g fresh weight.

**Table 4:** Analysis of variance (ANOVA) *p*-values and regression coefficients for the second-order response surface model in terms of the second optimization approach.

Source of Variance	*p*-Value	Regression Coefficient
Constant	0.000	19.528
Temperature	0.000	4.325
Solvent ratio	0.000	2.75
Sonication time	0.000	2.072
Temperature × Temperature	0.000	−1.661
Solvent ratio × Solvent ratio	0.000	−1.287
Sonication time × Sonication time	0.000	−1.971
Temperature × Solvent ratio	0.840	0.038
Temperature × Sonication time	0.000	1.842
Solvent ratio × Sonication time	0.070	−0.37
Lack-of-fit	0.182	

**Table 5:** Optimization parameters and their levels used in the central composite designs of the surface response method.

	Level				
Optimization Parameter	Low-Axial (−1.68)	Low (−1)	Center (0)	High (+1)	High-Axial (+1.68)
1st Optimization approach					
% Ethanol in water	0	20.24	50	79.76	100
Extraction time (h)	24	62.86	120	177.14	216
Solvent: solid ratio (*v:w*)	10	16.07	25	33.92	40
2nd Optimization approach					
Temperature (°C)	20	28.1	40	51.9	60
Solvent: solid ratio (*v:w*)	4	5.62	8	10.38	12
Sonication time (min)	10	20.12	35	49.88	60

## Data Availability

Not applicable.

## Sample Availability

Samples of grape seeds are available from the authors.

## Abbreviations

CCD	central composite design
GS	grape seeds
GSP	grape seed powder
MC	methylcellulose
mg CE/g FW	mg catechin equivalence/g fresh weight
PAC	proanthocyanidins
RSM	response surface method
s:s ratio	solvent: solid ratio
